# Evaluation of the NHS active 10 walking app intervention through time-series analysis in 201,688 individuals

**DOI:** 10.1038/s41746-025-01785-x

**Published:** 2025-08-06

**Authors:** Dharani Yerrakalva, Samantha Hajna, Soren Brage, Simon J. Griffin

**Affiliations:** 1 Department of Public Health and Primary Care, University of Cambridge School of Clinical Medicine, Cambridge, UK; 2 Department of Health Sciences, Faculty of Applied Health Sciences, Brock University, St Catharines, ON Canada; 3 MRC Epidemiology Unit, University of Cambridge, School of Clinical Medicine, Cambridge, UK

**Keywords:** Lifestyle modification, Preventive medicine

## Abstract

Despite widespread interest in integrating mobile health apps into primary care to prevent and manage physical inactivity-related health conditions, the effectiveness of these apps remains unclear. We quantified the effects of Active 10 (a goal setting and self-monitoring app developed by Public Health England) on brisk and non-brisk walking using a single-group interrupted time-series analysis of individual-level data collected between July 2021 and January 2024. Among Active 10 users (*n* = 201,668 l; 51.4 ± 14.4 years; 75.4% women) brisk and non-brisk walking increased by 9.0 (95% confidence interval (CI) 8.9, 9.1; 73% above baseline) and 2.6 min/day (95% CI 2.4, 2.8; 9% above baseline), respectively, on the day of app download. Post-download, brisk and non-brisk walking decreased by 0.15 (95% CI −0.17, −0.13) and 0.06 (95% CI −0.08, −0.03) min/day/month, respectively, but remained above baseline. Our findings suggest that Active 10 may be effective in facilitating increases in brisk and non-brisk walking.

## Introduction

The impact of population physical inactivity is seen in healthcare systems through its significant clinical, social, and financial burden.[1] Physical inactivity is associated with higher rates of cardiovascular disease, type 2 diabetes, cancers, dementia, depression and premature mortality^1–4^. Globally, up to 3.9 million excess premature deaths per year, and health care costs of 27 billion US dollars per year are attributable to physical inactivity^5,6^. Levels of inactivity are high among English adults (26% and 11% achieving <30 mins/week and 30−149 mins/week of activity, respectively)^7^. Creating an effective programme for improving population physical activity (PA) levels therefore represents an important public health goal for governments and national health services. However, few interventions designed to increase PA among adults have demonstrated sustained changes beyond 12 months^8–11^. The UK’s NICE guidelines recommend that brief physical activity advice is delivered in primary care^12^ but this is currently very challenging in this increasingly time-constrained setting. Consequently, there is a need to consider more innovative and scalable strategies that will encourage people to move more and sustain these improvements for longer.

Mobile health (mHealth) apps have been integrated into England’s public health approach to prevent and manage physical inactivity-related health conditions in recent years^13,14^. Apps allow tailored feedback, goal setting opportunities, and activity reminders throughout the day. Unlike traditional PA interventions (e.g., professional-led, group based), they are scalable with the potential to reach large segments of the population, and do not require input from the increasingly stretched health and social care workforce. If a large-scale intervention could lead to small individual-level changes, this has the potential to shift population-levels of PA and consequently lead to reductions in morbidity and premature mortality. However, the evidence base for the effectiveness of such innovations has not kept pace with their introduction. Mhealth PA interventions appear to be effective in the general adult population^15^ over short periods of follow-up, but it is unclear whether effects are sustained in the longer term.

Active 10, a mobile phone app introduced in 2017 by Public Health England to increase brisk walking levels, is one such mHealth intervention. Walking is the most common form of activity reported by English adults^7^, and therefore is an obvious target activity. Over the last year, Active 10 has been used by 82,825 users per month, with over 1.5 million total downloads since its introduction. Though recommended through public health campaigns in England, it has not been fully integrated into routine health care services. If effective, its use could be recommended to all patients identified in primary care as not sufficiently physically active as a means of increasing individual and population-levels of PA. The app has previously been described^16^ and data from early users have been presented^17^, however there has been no formal evaluation of its effectiveness. To fill this gap in knowledge, we aimed to evaluate whether Active 10 is effective in assisting users to increase their brisk and non-brisk walking levels, and to examine if any observed changes varied according to age, sex, baseline walking levels, total duration of engagement and engagement with different app features.

## Results

A total of 308,028 individuals accumulated at least one day of activity data between July 2021 and January 2024. Of these users, 75%, 65%, 35%, 21% and 6% continued to use the app beyond two weeks, four weeks, six months, 12 months and 24 months, respectively. Age and sex information was only available for 44.9% and 60.9% of individuals, respectively (Supplementary Table 3). Of those who provided this information, 76.6% were female and the mean age was mean age 48.3 (SD 19.8).

Valid data as per our inclusion criteria (≥5 days of pre-download data and ≥28 days of post-app download data) were available from 201,668 individuals. Of these included users, 75%, 55%, 32% and 10% continued to use the app beyond 3 months, 6 months, 12 months and 24 months, respectively, representing a total of 10.5 million-person-days. The app was used for a median of 6.6 months (range 1-30). The average number of days of pre-download data per individual were 6.1 (SD 0.5). Age and sex information was only available in 52.4% and 66.1% of individuals, respectively (Table 1). Of those who provided this information, 75.4% were female and the mean age was 51.4 years (SD 14.4). The least active quartile of individuals at baseline accumulated <2 mins/day of pre-download brisk walking, and the most active quartile accumulated ≥22 mins/day of pre-download brisk walking (equivalent to UK Guidelines)^18^. The distribution of users opening the app <1 times/week, 1- < 2 times/week, 2- < 4 times/week, 4- < 6 times/week and ≥6 times/week was 60%, 21%, 15%, 4% and 1%, respectively. 73% of users used target setting, 4% downloaded the widget, 8% read ≥ two health-related articles and 4% read ≥ five health-related articles.71.7% were iOS users. This is likely due to iOS devices more reliably allowing the app to access and store a greater number of days of historical activity data compared to Android devices. The app was downloaded most frequently in summer (39.3% of downloads), followed by winter (24.4% of downloads).

**Table 1:** Characteristics of included individuals (*n* = 201,668)

Variable		Frequency	Percentage of total (%)	Percentage of those reporting (%), *n* = 96072
**Sex**	Women	100,554	49.9	75.4
	Men	32514	16.1	24.4
	Prefer not to say	281	0.1	0.2
	Missing	68319	33.9	-
**Age**	<18	775	0.3	0.8
	18−29	7794	3.9	8.1
	30−39	12339	6.1	12.8
	40−49	17990	8.9	18.7
	50−59	26459	13.1	27.5
	60−69	21699	10.8	22.6
	70−79	8255	4.1	8.6
	≥80	761	0.4	0.8
	Missing	105,596	52.4	-
**Operating system**	Android	57,133	28.3	-
	iOS	144,535	71.7	-
**Baseline daily brisk walking levels (minutes)**	≤5	58,887	29.2	-
	5−10	33,074	16.4	-
	>10−15	26,620	13.2	-
	>15−20	28,435	14.1	-
	>20	54,652	27.1	-
**Season of app download**	Winter	49,265	24.4	-
	Spring	36,371	18.0	-
	Summer	79,194	39.3	-
	Autumn	36,838	18.3	-
**Year of app download**	2021	71,996	35.7	-
	2022	71,993	35.6	-
	2023	57,476	28.5	-
	2024	203	0.1	-
**Duration of App Use (months)**	≤3	50,820	25.2	-
	>3−6	40,535	20.1	-
	>6−12	45,779	22.7	-
	>12−24	43,762	21.7	-
	>24	20,772	10.3	-
**App opening frequency**	<1/week	119,945	59.5	-
	1- < 2	42,048	20.9	-
	2- < 4/week	29,267	14.5	-
	4- < 6/week	7,886	3.9	-
	≥6/week	2,522	1.2	-
**Target set**	Yes	146,190	72.5	-
	No	55,478	27.5	-
**Widget downloaded**	Yes	8,354	4.1	-
	No	193,314	95.9	-
**Read ≥ two health articles**	Yes	16,711	8.3	-
	No	184,957	91.7	-
**Read ≥ five health articles**	Yes	7,089	3.5	-
	No	194,579	96.5	-

Mean age 51.4 (SD 14.4). Median age 53 (IQR 41–62).

**Fig. 1:**
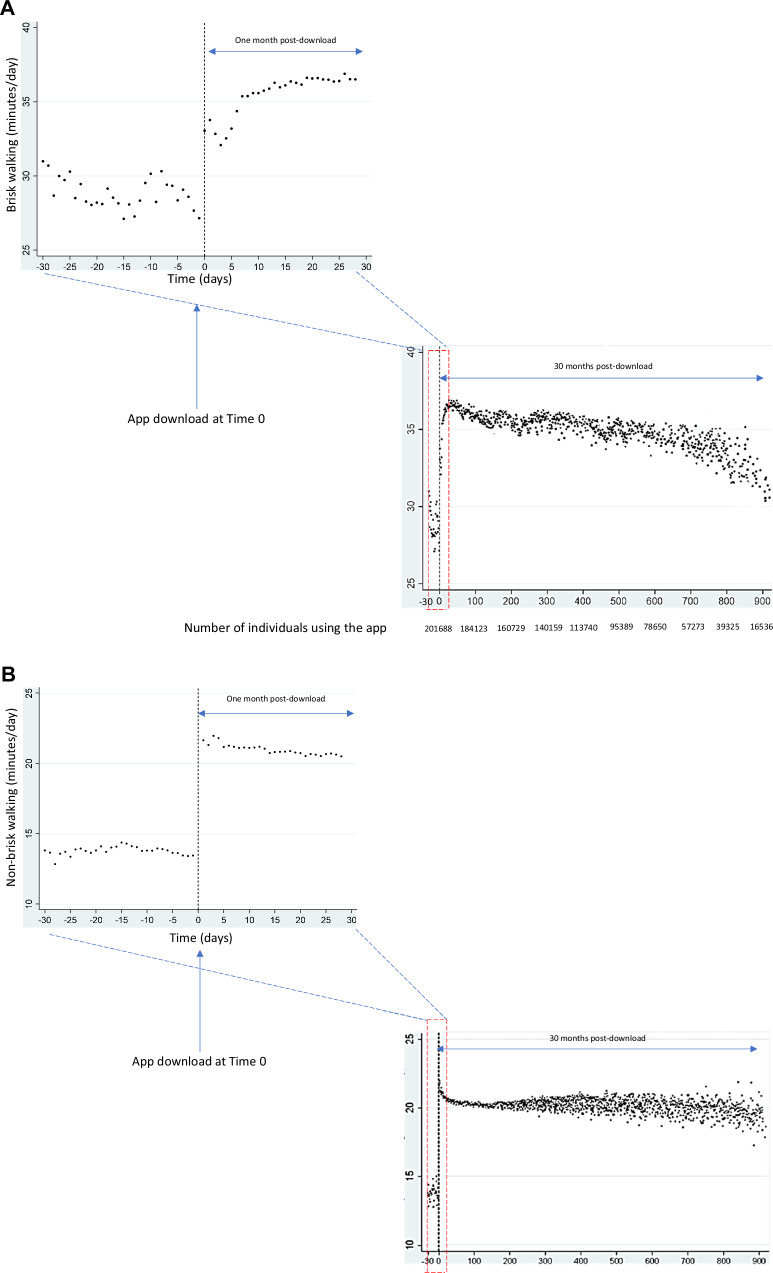
Time series to assess the effect of Active 10 use on change in daily brisk and non-brisk walking levels. The app was downloaded at time zero. Panel (**a**) shows daily brisk walking and panel (**b**) shows daily non-brisk walking time. Within each panel, the first graph shows how daily walking time changes from the one month pre-download through the one month post-download only. The second graph shows how daily walking time changes from the one month pre-download through the whole post-download period. All analyses were adjusted for seasonality. **a** Daily brisk walking levels (**b**) Daily non-brisk walking levels.

At baseline pre-download, individuals spent 12.3 mins/day (95% CI 12.1, 12.5) in brisk walking and 30.4 mins/day (95% CI 30.2, 30.6) in non-brisk walking (Table 2, Fig. 1). Prior to download, brisk and non-brisk walking were stable (pre-download trend change was 0.20 mins/day/month, 95% CI −0.4, 0.7 and −0.1 mins/day/month, 95% CI -0.9, 0.8 for brisk and non-brisk walking, respectively). On the first day the app was downloaded, brisk walking increased by 9.0 minutes/day (95% CI 8.9, 9.1), or 73% above baseline, non-brisk walking increased by 2.6 minutes/day (95% CI 2.4, 2.8), or 9% above baseline. Subsequently over the post-download period, there was a 0.15 min/day per month reduction in brisk walking (95% CI −0.17, −0.13) and 0.06 min/day per month reduction in non-brisk walking (95% CI −0.08, −0.03).

**Table 2:** Time series to assess the effect of Active 10 app on daily walking time

There was no pre-download trend of change in brisk (0.20 mins/day/month, 95% CI −0., 0.7) or non-brisk walking (−0.1 mins/day/month, 95% CI −0.9, 0.8) in the unstratified analysis. Similarly, there was no trend within any of the stratified analyses. All analyses were adjusted for seasonality. If the *p*-value from the cross-model Wald test for difference in intervention effect (post-download change on Day 1) between the models with and without the interaction term including the variable of interest was <0.05, it is denoted by red font (*p* values can be found in Supplementary Table 4).

The post-download trend of decline in brisk walking was initially more steep across the first month (1.2 mins/day/month, 95% CI 0.9, 1.5) (Fig. 1) before levelling off to a gentle decline. The post-download trend in non-brisk walking was initially a steep increase in the first month (3.5 mins/day/month, 95% CI 3.2, 3.8), followed by a gradual decline over the rest of the post-download period. At the end of the post-download period (30 months), brisk and non-brisk walking levels remained 4.5 and 0.8 mins/day (i.e., 37 and 0.03%, respectively) above pre-download levels.

The increase in brisk walking observed immediately post-download was greater for older compared to younger adults (e.g., 9.1 minutes for 50–59 year olds (95% CI 8.9, 9.3) and 8.0 min for 18–29 year olds (95% CI 7.6, 8.4)) (Table 2). The subsequent decline in post-download brisk walking was larger for older compared to younger age groups (e.g., 60–69 years old: -0.18 mins/day/month, 95% CI −0.20, −0.15 vs 18-29 years: -0.12 mins/day/month, 95% CI −0.14, −0.11). Similarly, the decline in post-download non-brisk walking over time was larger for older adults compared to younger adults. Immediately post-download, there was a greater increase among men than women in brisk (men: 10.9 mins/day, 95% CI 10.4, 11.3 vs women: 8.5 mins/day, 95% CI 8.3, 8.6) and non-brisk walking (men: 3.8 mins/day, 95%CI 3.2, 4.4 vs women: 1.8 mins/day, 95% CI 1.5, 2.0). Over time, the decline in post-download non-brisk walking was larger for men than women. The results for those that did not report age or sex were similar to those for the total sample. There were no significant differences for all brisk and non-brisk walking results between operating systems.

The immediate post-download increase in brisk and non-brisk walking was greater for those with greater baseline walking levels (e.g.,≤5 mins/day group: 5.8 mins (95%CI 5.4, 6.2) vs >20 mins/day group: 12.1 mins (95% CI 11.2, 13.0)). The decline in post-download brisk walking over time was steeper for those who were least active at baseline compared to other activity groups (e.g. <5 mins/day group: −0.21 mins/day/month, 95% CI −0.24, −0.18 vs >20 mins/day group: -0.12 mins/day/month, 95% CI −0.15, −0.09).

The baseline pre-download brisk and non-brisk walking levels were similar for users that initiated the program in different seasons. There were also no significant seasonal differences between post-download increases or trends for both brisk and non-brisk walking. There were no significant differences for all brisk and non-brisk walking results between calendar years.

The immediate post-download increase in brisk walking was greater for those who used the app for longer ( < 3 months:7.8 mins (95%CI 7.6, 8.0) vs 6–12 months: 9.9 mins (95%CI 9.7, 10.2). There were no differences for non-brisk walking. The decline in post-intervention brisk walking was steeper for individuals who used the app for >3–6 months compared to longer term users (e.g., >3–6 months: −0.17 mins/day/month, 95% CI −0.19, −0.15 vs >12–24 months: -0.12 mins/day/month, 95% CI −0.18, −0.06).

The immediate post-download increase in brisk and non-brisk walking was greater for groups who opened the app more frequently (e.g., brisk walking, <1 opening/week, 7.0 mins/day, 95% CI 6.9, 7.1 vs 2– < 4 openings/week, 9.9 mins/day, 95% CI 9.7, 10.1). The decline in post-intervention brisk walking over time was less steep for those who opened the app more frequently (e.g., <1 opening/week −0.15 mins/day/month, 95% CI −0.18, −0.12, vs 4- < 6 openings/week −0.06 mins/day/month, 95% CI −0.08, −0.03).

Those who had set targets had a greater immediate increase in brisk and non-brisk walking than those that did not (brisk walking, target set: 9.2 mins/day, 95% CI 9.0, 9.3 vs no target set: 7.0 mins/day, 95% CI 6.5, 7.4; non-brisk walking, target set: 6.3 mins/day, 95% CI 5.6, 6.9 vs no target set: 2.5 mins/day, 95% CI 2.3, 2.8). Compared to those who downloaded the widget, those who did not had a greater subsequent decline in brisk walking (download: −0.06 mins/day/month, 95% CI −0.12, −0.03 vs no download: −0.15 mins/day/month, 95% CI −0.17, −0.13). Furthermore, those who downloaded the widget had a greater immediate increase in non-brisk walking than those who did not (download: 2.8 mins/day, 95% CI 2.6, 3.0 vs no download: 2.1 mins/day, 95% CI 1.9, 2.3)). Those who had read ≥2 health articles had a higher immediate increase in brisk walking than those who had not (read: 10.2 mins/day 95% CI 9.7, 10.8 vs not read: 8.8 mins/day, 95% CI 8.6, 8.9). The results were similar for those who had read ≥5 health articles versus not. Those who read ≥5 articles had a lower decline in non-brisk walking over time (read: −0.03 mins/day/month, 95% CI −0.05, −0.02 vs not read: −0.06 mins/day/month, 95% CI −0.08, −0.05).”

## Discussion

We report the effectiveness of a mobile app intervention to promote walking, implemented through English national public health campaigns from July 2021 to January 2024, on a large population of predominantly UK adults. We found that the app appeared to be effective, with an immediate improvement in brisk (9.0 mins/day, 73% above baseline) and non-brisk walking time (2.6 mins/day, 9% above baseline). For those who continued to use the app, the effect on brisk walking and non-brisk walking levels was attenuated but was maintained above pre-intervention levels even at 30 months.

During the first month, the initial steeper decline in brisk walking coincided with an initial increase in non-brisk walking which could be explained by an intensity ‘recalibration’ following the initial intervention effect. Some of the initial new brisk walking may be being replaced with slower (non-brisk) walking which still represents a positive change from the pre-intervention period. Those who used the app for longer, opened the app more frequently, set themselves targets, and read health-related articles within the app had a greater immediate increase in brisk walking. Additionally, the post-download decline in brisk walking was lower for those who used the app for longer, opened the app more frequently, and for those who downloaded the widget. The app was most frequently downloaded in the summer, followed by winter. Summer engagement is likely attributed to better weather in the UK at this time of year, and winter engagement may be due to New Year’s health resolutions. Regardless, the apparent impact of the app did not vary significantly by season of download. These findings give us an insight into the behaviour change techniques that might mediate effects of the app, and indicate important features for future interventions.

The level of retention of users compares favourably to other apps^19^ with 21% of all users continuing to use the app beyond a year. Of those choosing to report their personal characteristics, we found that more women than men used the app (75%) and a higher-than-expected proportion of users were older adults (32% ≥60 years old). This goes against the narrative that older adults may be subject to digital exclusion^20^ although one would expect this issue to diminish as smartphone usage continues to increase at the population level, in particular in older age groups^21^. The app was designed to target inactive adults, and 75% of users were not meeting UK activity guidelines at baseline^18^. We report here that although the app was more effective in increasing walking behaviour among those who were more active at baseline, it was still effective in those who were initially inactive.

Though previous PA interventions have been associated with short-term effects^10^, the findings here are important given the sparsity of evidence for long-term effects of PA interventions and interventions at the population-level. Self-guided interventions are potentially more scalable, but a recent meta-analysis by Stavric et al.^22^ has shown that such PA interventions have led to only small changes in PA (Standardised Mean Difference (SMD) 0.2, 95% CI 0.1, 0.3), and changes have not persisted beyond a year (SMD 0.3, 95% CI − 0.2, 0.9). Similarly, meta-analyses of trials assessing effectiveness of mobile app interventions in adults^23,24^ and older adults^25^ have found that they have the potential to be effective for behaviour change in the short-term, especially when paired with an activity tracker such as a smart watch, but data from longer-term follow-up are sparse.

Recently, Yao et al.^26^ reported on findings from a population-level intervention in Singapore, in which all residents were offered a step counting programme (app with or without a paired wearable PA tracker and financial rewards, *n* = 421,388). The median duration of engagement was 74 days, and they reported a mean daily step count increase of 934 steps/day (95% CI 916,952). This study corroborates the findings from our UK-based study that PA interventions involving apps are scalable and effective. Our study goes beyond Yao et al. in providing evidence of longer-term effectiveness of such interventions, albeit among the minority whose use of the app was sustained. This strengthens the case for public health interventions using apps without financial incentives. Yao et al. did find that use of an activity tracker appeared to be more effective in activity change versus a smartphone alone, and this could be a future extension to increase the effectiveness of Active 10; however, differential measurement bias between phones and wearables over time may also explain the apparent greater effect.

A recent meta-analysis by Gasana et al.^9^ reported on the long-term effects of PA interventions on objectively-measured PA outcomes. They found four studies examining PA interventions only, and a further two studies examining mixed diet and exercise interventions. Their meta-analyses included studies assessing mixed diet and PA interventions, but found a significant effect of interventions on moderate-to-vigorous PA (MVPA) at 24 months (four studies, SMD: 0.18 95% CI 0.07, 0.29, only two studies were PA-only) and at 36 to 48 months (three studies, SMD: 0.16 95% CI: 0.09, 0.23, only one study was PA-only). This equates to a difference of 21–25 min/week of MVPA at 24 months (assuming SD of 118–140 min at 24 months). This is comparable to the effect sizes we found: at 18 months, time spent in brisk walking was 6.3 min/day (44.1 min/week) above baseline, and at 30 months, brisk walking was 4.5 min/day (31.5 min/week) above baseline.

To our knowledge, there is only one PA-only intervention study that has demonstrated effectiveness beyond 12 months^8,27,28^. This was Harris et al.^8,27^ (PACE-UP, PACE-LIFT) in which UK participants were followed up for four years after a 12-week pedometer-based walking trial, including both nurse-supported and postal pedometer arms (pedometer, handbooks, PA diaries). In PACE-UP, (mean age 59, 64% female, 67% retention) and PACE-LIFT (mean age 67, 53% female, 76% retention), they found that individuals in the nurse-led and postal pedometer arms continued to have 28 (95% CI 7,49) and 24 mins/week (95% CI 3,45) more time in MVPA bouts of >10 minutes compared to the control group. The intervention we report here delivers a similar effect size to the interventions in PACE-UP and PACE-LIFT but is potentially more scalable given its independence from health professional support and a separate wearable device.

We found user retention to be 35% at 6 months and 21% at 12 months. Of all health and fitness app installs worldwide, day 30 retention rates stand at 2.8%^19^. The favourable Active 10 retention rate is likely to be reflective of it being free and endorsed by English Public Health departments, though future work to understand how retention may be optimised would be useful.

Our findings of female and older age preference are in keeping with other population-level app studies^26,29^, with the caveat that age and sex data were only available in a subsample. Yao et al.^26^ found that 58% of app users were female in the population-wide Singaporean intervention, and 11.3% were aged >60 years. Pontin et al.^29^ examined the socio-demographic determinants of physical activity app usage, looking at data from users of the walking incentive app, Bounts, and also found a predominance of female users (77%). This has important implications for future design of such interventions, which could be explored in qualitative work (e.g., it would be important to explore how men can be encouraged to use PA apps).

The use of the Active 10 app led to an initial improvement in brisk walking of 9 mins/day, equating to 63 mins/week, and it remained at 4.5 mins/day above baseline at 30 months post-download in those who continued to use the app (31.5 mins/week). An impact on activity such as this, if replicated and sustained, could potentially lead to clinically significant benefits at the population level e.g., on risk of cardiovascular events and premature mortality. In their analysis of observational data, Garcia et al.^4^ performed a meta-analysis of large prospective studies to estimate the dose–response associations between non-occupational physical activity and several chronic disease and mortality outcomes in the general adult population. They found that undertaking at least 75 mins/week of MVPA (or 11 mins/day), half of what is currently recommended in UK guidelines, would prevent 10% of premature mortality, 5% of cardiovascular morbidity and 3% of cancers. Further, 4-year follow-up of the PACE-UP RCT found that the primary care pedometer-based walking intervention led to fewer new cardiovascular events and fractures (Cox hazard ratios for time to first event for interventions versus controls for nonfatal cardiovascular were 0.24 (95% CI 0.07–0.77), total cardiovascular events 0.34 (95% CI 0.12–0.91) fractures 0.56 (95% CI 0.35–0.90, *p* = 0.02)). In the context of the current literature further RCT-level evidence is needed to determine whether an intervention such as Active 10 has longer term health benefits.

We utilised data from a large sample, giving us greater power to assess overall effectiveness and potential sub-group effects. As data were stored in an anonymised form, and were collected without needing to attend a healthcare facility, this dataset may reflect a more socioeconomically and ethnically diverse population than would normally be found in more selective RCT samples^30^. Our approach represents a pragmatic solution to generating evidence on the effectiveness of a large-scale app intervention given that an RCT of comparable size and length of follow-up would be associated with significant cost, logistical challenges and potential Hawthorne-like effects.

Several potential limitations should also be noted. First, given that this intervention was not delivered under randomised-control conditions, we cannot rule out that observed effects are in part due to co-interventions or time-varying confounding. However, we consider it highly unlikely that such external factors would account for the effect sizes that we observed at exactly the time the Active 10 app was downloaded for individuals over the duration of the study period (i.e., a changing external factor coinciding with app download for some individuals will affect baseline data for individuals downloading the app later in the study). We accounted for potential impact of seasonality through autocorrelation testing and adjustment of analyses for seasonality. Second, we did not have data on walking levels from users once they stopped using the app, and therefore we do not know if users maintained their higher walking levels or if they returned to their baseline levels. Third, users self-selected to download Active 10 so our study population is likely to be different from the average population (e.g., healthier, more motivated). Further, our included sample was slightly older than the wider population of individuals that accumulated at least one day of activity data on Active 10 and therefore our findings may not be generalisable to the entirety of the UK population and whole population effect sizes are likely to be smaller than we report.

Fourth, there are several potential sources of measurement error and bias. Smartphones measure walking reasonably well but do not accurately assess other common forms of PA such as cycling. We were therefore unable to detect compensatory behaviour change from one activity type to another; it is theoretically possible that some users may have switched from cycling to walking as a result of using the app but therefore not increased their overall activity level and with no net gain in health. Similarly, phones are not carried on the body all of the time in free-living conditions (e.g., left while carrying out indoor daily living activities such as cleaning) which may lead to the underestimation of total PA^31^, both pre- and post-download. It is also possible that where the phone is carried (e.g., purse vs pocket) could lead to differential measurement of activity and therefore bias^32^. Pre-download activity data could be under-estimated by change in user phone carriage and location following download (e.g., an individual starts to carry their phone on themselves more when they download the app) and therefore may lead to an overestimation of the initial effect of the app. A future study could include measurement of pre- and post-download levels with a separate activity monitor to assess measurement error and bias arising from differences in phone carriage location.

Fifth, the pre-intervention data were limited to five days due to privacy settings on mobile phones. Although greater pre-intervention data would have provided a more accurate estimate of pre-intervention walking levels, our analyses reassuringly indicated behavioural stability over the 5 days. Finally, stratified analyses by age and sex were limited since information was incomplete on these characteristics; such analyses highlight elements of the digital intervention that may need optimising for certain population subgroups. The results for those that did not report age or sex were similar to those for the total sample, suggesting that reporting of these characteristics are unrelated to physical activity. That said, we were able to provide evidence that the intervention was effective in reaching users at greatest need (the most inactive) at baseline; all groups improved and the least active had the most relative improvement.

Though the accuracy of the Active 10 app’s quantification of PA against other objective measures (e.g., wearable devices or stable isotopes) has not been documented in the literature, validation has been carried out of other PA apps which use very similar measurement methods (e.g., Apple Health app) indicating that they are moderately accurate^33–35^. Coupled with the fact that some measurement error will be the same at baseline and follow-up and thus accounted for in a pre-to-post intervention evaluation such as ours, we can therefore be reasonably confident that measurement error would not fully account for the effects that we observe.

We recommend future studies to be designed in light of these limitations, examining the effectiveness of a multi-faceted national app-based interventions including features which have shown to be effective in smaller samples (e.g., linkage to a wearable device, rewards, personalisation) under randomised-control conditions, collecting a wider and fuller range of sociodemographic variables, and extending follow-up in large samples to also allow evaluation of effects on health outcomes. Further work should also concentrate on how users might be retained given the potential benefit of long-term use; this might include learning from other industries that gamify technologies and utilise financial incentives to keep users engaged. Finally, our work and similar studies should contribute towards future cost-effectiveness analyses.

Though UK NICE guidelines currently recommend the delivery of brief physical activity advice within primary care settings, this does not take into account clinician’s ’time to treat’. Johansen et al.^36^ predict that implementation of the NICE physical activity guideline would require 15% (167 of 1128 h) of the GP’s yearly total face-to-face time with patients, which is unlikely to be feasible. Apps such as Active 10 could provide a mechanism to support delivery of such guidelines, making their implementation possible in real-world settings.

The Active 10 brisk walking app has been successfully delivered through English Public Health campaigns and has demonstrated large reach and acceptability. It appears to be effective in enabling changes in walking levels. We give evidence to support the ongoing commissioning of this type of digital public health intervention, and consideration of integration into NHS systems e.g., data synchronisation with primary care systems, so that healthcare professionals can endorse public health recommendations, monitor progress, and adapt other elements of the care pathway to enable greater degree of personalised medicine.

## Methods

We accessed anonymised walking data collected by the Active 10 app from 308,028 of its users between July 2021 and January 2024 via the Department of Health & Social Care (DHSC). Users of the app consented to the collection of anonymised data and the sharing of these data for research purposes^37^. On first opening the app, all users were asked to agree to the terms and conditions of the privacy policy which outlined this. We (the University of Cambridge) and DHSC agreed a data sharing and collaboration agreement in order to access these anonymised data. To be included in this study, individuals must have downloaded and opened Active 10, have ≥5 days of pre-download walking data (extracted automatically, with consent, by Active 10 from users’ mobile devices), and have ≥28 valid days of walking data recorded by Active 10 immediately following download of the app.

The intervention comprised of Active 10, an app that was designed, promoted, and delivered through English Public Health campaigns initially by Public Health England and then by the Office for Health Improvement and Disparities in the DHSC between 2017 to present. Active 10’s development was underpinned by behaviour change theory [13] and user feedback, and its functions have been mapped to behaviour change techniques (BCTs) (Supplementary Table 1). The design and roll-out of the Active 10 app has previously been described in detail elsewhere^16,17^. In brief, Active 10 tracks user activity levels using multiple in-built phone sensors including accelerometers and gyro sensors. The user aims to achieve a target of one to three brisk walking bouts per day, with a bout being defined as 10 min of brisk walking (referred to as an ‘Active 10’). We describe the intervention here according to the Tidier intervention checklist^38^ (Supplementary Table 2).

**Fig. 2:**
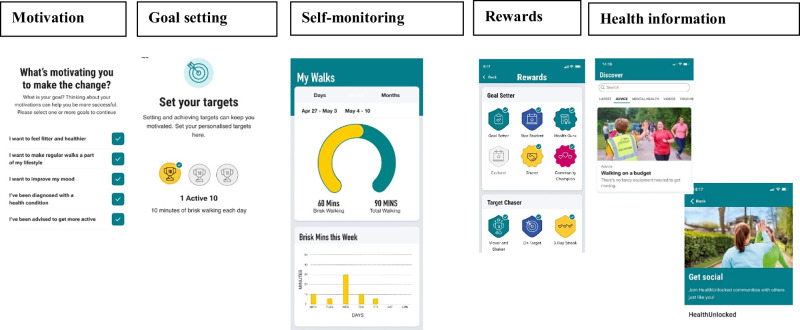
Active 10 intervention components. This figure demonstrates functionality of the app through screen shots. There are screenshots to demonstrate some of the behaviour change techniques used by the app (motivation, goal setting, self-monitoring, rewards and health information).

Active 10’s messaging is based on the theory that habit formation and persistence are more likely if change is small and achievable. It allows users to set motivating factors (e.g., ‘I want to feel fitter’, ‘I want to improve my mood’), set goals (one to three sets of ‘Active 10 s’, with the offer of higher targets if achieving three regularly) and shape their knowledge through health information articles viewed in the app (Fig. 2). It also allows users to earn rewards, monitor progress, and engage with social support (Active 10 forum, where users can post progress updates, encouragement and questions). Users also have an option to download a widget which shows daily brisk and non-brisk walking levels on the home screen.

The majority (93%) of Active 10 app downloads have been on UK registered devices. As the app is only available in UK app stores, the remaining 7% of users are likely people that live in the UK with a phone that was originally bought/setup in another country.

Data made available to be analysed was from users between July 2021 and January 2024, as historical data was only in that period.

Descriptive characteristics were limited to age and sex. Sex was coded as male, female, or not reported. We defined valid age as >5 years and <100 years. In earlier versions of the app, users were not asked to provide any demographic information in order to make initial set-up simpler and therefore maximise the number of individuals who completed onboarding and went on to use the app. In later versions, users were given the option to report their age and sex. Some users also opted not to share this information.

We utilised pre-download walking level and post-download walking level data. Following download, the app requested permission from the users to access their historical walking data. Dependent on the restrictions of their device’s operating system privacy protocols, these historical data were limited to the previous 5 to 30 days. Using these data, we generated daily brisk and non-brisk walking time (mins/day) variables. Baseline objective walking levels were calculated from the mean brisk walking time in mins/day across all the days of pre-download walking data. We generated categories of baseline walking levels (≤ 5, >5–10, >10–15, >15–20, >20 minutes/day).

The main outcome variables of interest were post-download daily brisk and non-brisk walking time (mins/day). For a walking minute to be registered, at least one step needed to be counted in each quarter of the minute (i.e., every 15 seconds). Active 10 utilised walking cadence (steps/minute) to estimate intensity of walking. Walking intensities at <100 and ≥100 steps/minute were used to define non-brisk and brisk walking, respectively.

Data were collected and categorical variables created for variables related to the nature of app utilisation. These included i) total duration of app use ( ≤ 3 months, >3–6 months, >6–12 months, >12–24 months, >24 months) ii) app opening frequency (mean number of times the app was opened per week; <1 times/week, 1- < 2 times/week, 2- < 4 times/week, 4- < 6 times/week, ≥6 times/week), iii) target setting (user set reasons for behaviour change in onboarding; Yes vs No), iv) read ≥two health-related articles (Yes vs No), v) read ≥five health-related articles (Yes vs No), and vi) widget download (Opted to have icon on phone interface with display of daily brisk and non-brisk minutes; Yes vs No).

We calculated descriptive statistics for all who registered for the app (age, sex, operating system and year of download). We calculated descriptive statistics for users included in the analysis (age, sex, and baseline walking levels, app opening frequency, mobile phone operating system, season of app download, year of download, pre- and post-download spent in non-brisk and brisk walking time in minutes/day).

We performed interrupted time-series analyses (ITSA) to examine whether there was a step change and a trend change in brisk and non-brisk walking levels following app download. We used individual-level data to perform a single-group ITSA with panel data (utilising XTITSA command). We tested for autocorrelation using the actest function (up to lag 365 to assess for seasonal effect; if activity correlates with season of previous year) and found no autocorrelation at any lag. We generated continuous cosinor variables for spring (sin(2*π*day of year of measurement/365.25)) and winter (cos(2* π*day of year of measurement/365.25)). Analyses were adjusted for seasonality using these variables, since app use started at varying times of the year and the UK has a temperate climate which can affect activity levels^39,40^); this adjustment aims to remove such effects and reduce noise in the evaluation of the intervention effect. We also ran the ITSA using data from the first 30 days post-download only, to examine whether the step change and trend change results would be different compared to the analysis utilising the whole post-download period.

We ran ITSA, stratified by total duration of app use, to examine whether the step change and trend change results would be different depending on length of engagement. We carried out stratified analyses by app engagement (app opening frequency, target setting, reading health articles, widget download), baseline walking levels, age groups (18–29, 30–39, 40–49, 50–59, 60–69, 70–79, ≥80, not reported), sex (male, female, not reported), operating system, season of download (winter, spring, summer, autumn) and calendar year (2021, 2022, 2023). For each stratum, we ran an ITSA within the stratum, and compared the 95% CI of the effect estimates. To test whether there was a difference in intervention effect between strata, we ran a model with and without the stratifier of interest as an interaction term, then tested the two models against each other (cross-model Wald’s test utilising xtgee and margins commands). We conducted our analyses using Stata 17 (College Station, TX: StataCorp LP).

## Supplementary information

Supplementary Tables

## Data Availability

The data used here is owned by the DHSC, and applications for data for further analyses must be made to the DHSC directly. The minimal dataset and code needed to interpret, replication and build on methods and findings in this article can be accessed on application to the authors.

## Code availability

The code can be made available on application to the authors.
