# Supplementary material for: Evaluation of the NHS active 10 walking app intervention through time-series analysis in 201,688 individuals
Source: NPJ Digit Med. 2025 Aug 6;8:441. doi: 10.1038/s41746-025-01785-x (PMC12328748; doi:10.1038/s41746-025-01785-x)
Supplement: Supplementary file 1 — Supplementary Tables [file 41746_2025_1785_MOESM1_ESM.pdf]

Supplementary Table 1: TiDieR Checklist

**Brief Name**	• Active 10
**What is it**	• It is a walking app centred around goal setting and self-monitoring of brisk walking time. The user aims to achieve a target number of brisk walking bouts per day, where a bout is 10 brisk walking minutes (an 'Active 10').
**How it works:**	• tracks the users' activity levels using in-built phone sensors (accelerometers and gyro sensors)<br>• measures brisk and non-brisk walking minutes<br>• the user aims to achieve one to three bouts of 10 brisk walking minutes per day.<br>• The two key messages are: 1) walk 10 minutes briskly and 2) build on this with one or two more 'Active 10s' to achieve 30 minutes of activity a day.
**Theory central to the intervention**	• It is based on the premise that habit formation and persistence is more likely if change is small and achievable.<br>• It uses a number of BCTs (goal setting, target setting, shaping knowledge, earning rewards, monitoring progress, social support engagement
**How and where is it delivered:**	• It has been promoted during English Public Health Campaigns from 2017 to present (e.g., posters and commercials)<br>• It featured in a BBC documentary (The Truth About Getting Fit).<br>• It is assumed that a large proportion of users have self-downloaded the app through these campaigns and are likely not receiving additional support.<br>• It is accessible through Android phones, downloadable through the google play store, and Apple phones through the Apple apps store.
**When and how much:**	• We describe data from users of the app between from July 2021 to January 2024.<br>• How much the app is used is user-led, but it must be opened at least every five days.<br>• Users have an option to set daily prompts, and download a widget which shows brisk and non-brisk walking minutes on the home screen.
**Tailoring**	• If users set the target of achieving three Active 10s and achieved this, they were offered the opportunity to increase their target number of Active 10s.
**Monitoring App progress**	• The Department of Health & Social Care has used focus groups and user feedback through the app store to monitor the acceptability of the app, and decide on improvements.

Supplementary Table 2: Mapped Active 10 function to Behaviour Change Techniques (BCT) Codes

**BCT (code)**	**Active 10 function**
Goal setting (behaviour) (1.1)	Set on download and can update later.<br>Goal e.g. 'I want to feel fitter; I want to improve my mood).<br>Targets e.g., 1-3 Active 10s. If regularly achieving 3, then can set up to 6 Active 10s as target.
Review behaviour goals (1.5)/ Discrepancy between current behaviour and goal (1.6).	Main screen shows grey trophies for each target Active 10 achieved. These turn gold if achieved
Feedback and monitoring (2)	Records walking minutes and number of Active 10s achieved which can be reviewed in 'My Walks' tab.
Social Support (3)	Links to 'Health unlocked' (social network for health) and to organised walks through Rambler's association and Parkrun
Instructions on how to perform the behaviours (4.1)	Introduction: "Brisk walking is…" "Every minute counts" "Aim for 10 min or more a day".
Information on health consequences (5.1)	Prompts on benefits of exercise (e.g., improve your mood). Links to Better Health website.
Prompts/cues (7.1)	Can set prompt for reminder to walk at specific time of day. Prompts on benefits of exercise
Habit formation (8.3)	Can set prompt for reminder to walk at specific time
Material reward behaviour (10.2)	Can earn badges e.g., achieving Active 10s, doing this for consecutive days
Credible source (9.1)	NHS

Supplementary Table 3: Characteristics of included (n=201, 668) and excluded individuals (n=308,028)

For all who registered on the app, the mean age was 48.3 (SD 19.8) and the median was 50 (IQR 36-60). For those included in the analyses, mean age was 51.4 (SD 14.4) and the median age was 53 (IQR 41-62) (t-test for the difference between the mean ages; p<0.0001)

**Variable**		**All who registered (n=308, 028)**			**All included in analysis (n=201,668)**		
		**Frequency**	**Percentage of total (%)**	**Percentage of those reporting (%,)**	**Frequency**	**Percentage of total (%)**	**Percentage of those reporting (%)**
**Sex**	Women	144069	46.8	76.8	100554	49.9	75.4
	Men	42958	13.9	22.9	32514	16.1	24.4
	Prefer not to say	563	0.2	0.3	281	0.1	0.2
	Missing	120438	39.1	-	68319	33.9	-
**Age**	0-<18	2351	0.8	1.7	775	0.3	0.8
	18-<30	17841	5.8	12.9	7794	3.9	8.1
	30-<40	21852	7.1	15.8	12339	6.1	12.8
	40-<50	26831	8.7	19.4	17990	8.9	18.7
	50-<60	34437	11.2	24.9	26459	13.1	27.5
	60-<70	24896	8.1	18.0	21699	10.8	22.6
	70-<80	9268	3.0	6.7	8255	4.1	8.6
	≥80	829	0.3	0.6	761	0.4	0.8
	Missing	169723	55.1	-	105596	52.4	-
**Operating system**	Android	82859	26.9	-	57133	28.3	-
	iOS	225169	73.1	-	144535	71.7	-
**Season of download**	Winter	94565	30.7	-	49265	24.4	-
	Spring	57601	18.7	-	36371	18.0	-
	Summer	101957	33.1	-	79194	39.3	-
	Autumn	53905	17.5	-	36838	18.3	-
**Year of Download**	2021	103189	33.5	-	71996	35.7	-
	2022	108426	35.2	-	71993	35.6	-
	2023	90252	29.3	-	57476	28.5	-
	2024	6161	2.0	-	203	0.1	-

Supplementary Table 4: Cross-model Wald test for difference in intervention effect (post-download change on Day 1) between the models with and without the interaction term

Variable	Wald test for difference in intervention effect (post-download change on Day 1) between strata	
	**Brisk p value**	**Non-brisk p value**
**Sex**	<0.0001	<0.0001
**Age (years)**	<0.0001	0.62
**Baseline daily brisk walking levels (minutes)**	<0.0001	<0.0001
**Operating system**	0.29	0.51
**Season of download**	0.091	0.24
**Year of download**	0.21	0.15
**Duration of App Use (months)**	0.0000	0.10
**Number of app openings/week**	0.009	<0.0001
**Target setting**	<0.0001	0.0001
**Widget download**	0.089	0.0001
**Read ≥ two health articles**	0.012	0.65
**Read ≥ five health articles**	0.031	0.17
